# Lower serum prohepcidin levels associated with lower iron and erythropoietin requirements in hemodialysis patients with chronic hepatitis C

**DOI:** 10.1186/1471-2369-13-56

**Published:** 2012-07-07

**Authors:** Yasar Caliskan, Berna Yelken, Abdullah Ozkok, Numan Gorgulu, Halil Yazici, Aysegul Telci, Alaattin Yildiz

**Affiliations:** 1 Division of Nephrology, Department of Internal Medicine, Istanbul Faculty of Medicine, Istanbul University, Istanbul, Turkey; 2 Department of Biochemistry, Istanbul Faculty of Medicine, Istanbul University, Istanbul, Turkey

**Keywords:** Hemodialysis, Hepcidin, Iron metabolism, Inflammation, Hepatitis C, Ferritin

## Abstract

**Background:**

Patients with chronic HCV infection have increased liver iron. Recently identified protein hepcidin synthesized in the liver, is thought to be a key regulator for iron homeostasis and is induced by infection and inflammation. Lower erythropoietin and iron supplementation requirements were previously reported in HD patients with HCV infection. We investigated the association of prohepcidin with inflammation and iron parameters in HD patients with and without chronic HCV infection.

**Methods:**

Sixty patients (27 male, 33 female, mean age 50 ±15 years) on chronic HD were included. Parameters related to iron metabolism (ferritin, serum iron and total iron binding capacity (TIBC)), inflammation (hs-CRP, TNF-α and IL-6) and prohepcidin levels were measured. The response to treatment (erythropoiesis-stimulating agent (ESA) resistance index) was assessed from the ratio of the weekly erythropoietin (rhuEPO) dose to hemoglobin (Hb) per unit weight.

**Results:**

Serum prohepcidin levels of HCV positive patients (135 ± 25 ng/mL) were significantly lower than HCV negative patients [148 ± 18 ng/mL, (p = 0.025)]. Serum IL-6 levels of HCV positive patients were also significantly lower than HCV negative patients (p = 0.016). Serum prohepcidin levels were positively correlated with ferritin (r = 0.405, p = 0.001) and IL-6 (r = 0.271, p = 0.050) levels in HD patients. In the HCV positive group, serum prohepcidin levels significantly correlated with ferritin levels (r = 0.514 p = 0.004). In the HCV negative group, serum prohepcidin levels significantly correlated with serum IL-6 levels (r = 0.418, p = 0.027). In multiple regression analysis performed to predict prohepcidin in HCV positive patients, serum ferritin was found to be an independent variable (r = 0.28, p = 0.008).

**Conclusions:**

HCV positive HD patients have low levels of serum prohepcidin and IL-6 which might account for iron accumulation together with lower iron and rhuEPO requirements in these patients.

## Background

Hepatitis C virus (HCV) infection is the most common cause of chronic liver disease in the world and also common among chronic hemodialysis (HD) patients [1]. The prevalence of HCV infection in chronic HD patients has been estimated to be between 5 and 40% [2,3]. Patients with chronic HCV infection often have increased liver iron, however, little is known about the mechanism of iron accumulation in the liver [4]. Recently identified protein hepcidin, a 25-amino acid peptide hormone exclusively synthesized in the liver, is thought to be a key regulator for iron homeostasis and is induced by infection and inflammation [5-7]. In HD patients, compared to healthy controls, higher levels of prohepcidin and hepcidin associated with chronic inflammation were reported [7,8]. High levels of hepcidin in these patients were found to be related to an increased inflammation and resistance to recombinant human erythropoietin (rhuEPO) therapy [7,8]. Serum levels of prohepcidin, the precursor molecule of hepcidin, were found lower in patients with chronic HCV infection and concentrations of this molecule were found negatively associated with total iron stores in non-uremic patients with chronic HCV infection [9-11]. However, changes in hepcidin regulation have not been examined previously in HD patients with chronic HCV. We previously reported that lower erythropoietin and iron supplementation requirements in HD patients with HCV infection [12]. Impaired hepcidin regulation may play a role in alterations of iron metabolism in HD patients with HCV infection. In order to address this question, we investigated the association of prohepcidin with inflammation and iron parameters in HD patients with and without chronic HCV infection.

## Methods

### Patients

In our clinic, among 165 HD patients, 30 HD patients with chronic hepatitis C were selected according to inclusion criteria. After selection of the HCV positive patient group, the controls for each case were chosen from HCV negative patients, who were matched for age, sex and time under HD, in the same center. Sixty patients (27 male, 33 female, mean age 50 ± 15 years) on chronic HD for a mean time of 80 ± 51 months were included in the study. Of all study patients 30 had chronic HCV infection and the remaining 30 had negative anti-HCV test. A review of medical records including information on age, sex, baseline weight, time on HD treatment, the etiology of end stage renal disease (ESRD), serologic test results for viral hepatitis, cardiovascular disease risk factors, cardiac, thyroid and liver functions were undertaken. Twenty healthy subjects, aged between 27 and 57 years, were included as a control population. In HD study group, patients were receiving thrice weekly dialysis for a 4 hour period with a standard bicarbonate-containing dialysate bath, using biocompatible HD membrane (Polysulphone, FX-80 series, Fresenius, Germany). Blood flow rates ranged from 250 to 300 mL/min, while dialysate flow rate was kept constant at 500 mL/min. All HD patients were maintained at their target dry body weight and received an adequate dose of dialysis (double pool Kt/V≥1.4). In the HCV positive group, none of the patients had clinical (ascites, edema, jaundice, collateral circulation and splenomegaly) and radiological findings of hepatic cirrhosis. Patients in the HCV positive group were not receiving anti-viral therapy during the course of the study. The following patients were excluded from the study; (1) patients positive for hepatitis B virus surface antigen (HBsAg) (2) patients with previously diagnosed nonrenal cause of anemia other than iron deficiency (3) patients with an evidence of active or occult bleeding (4) patients who received blood transfusion within the past 4 months (5) patients with a history of malignancy, end-stage liver disease, or chronic hypoxia (6) patients with a history of recent hospitalization or infection requiring antibiotics within the past 4 weeks. Iron status was defined and iron requirement was supplied according to K/DOQI guidelines [13].

Our examinations of the patients conformed to good medical and laboratory practices and the recommendations of the Declaration of Helsinki on Biomedical Research involving Human Subjects. This study was approved by Ethical Committee of Istanbul School of Medicine. This study is registered to ClinicalTrials.gov, number NCT01272479.

### Laboratory data

Fasting serum samples were obtained in the early morning for biochemical studies. All biochemical blood samples were collected before the mid-week HD session in study group. Most laboratory values including complete blood cell counts and serum levels of urea nitrogen (BUN),creatinine, electrolytes, calcium, phosphorus, total protein, albumin, aspartate aminotransferase (AST), alanine aminotransferase (ALT), total cholesterol, high-density lipoprotein (HDL)-cholesterol, low-density lipoprotein (LDL)-cholesterol, very low-density lipoprotein (VLDL)-cholesterol, triglycerides, ferritin, iron and total iron binding capacity (TIBC), were measured by standard enzymatic procedures. Transferrin saturation (TSAT) was calculated as serum iron/total iron binding capacity. The response to treatment (erythropoiesis-stimulating agent (ESA) resistance index) was assessed from the ratio of the weekly erythropoietin (rhuEPO) dose to hemoglobin (Hb) per unit weight, yielding a continuous variable (weekly dose of rHuEPO/Hb/kg). High sensitive CRP (hs-CRP) (nephelometric method, Dade Behring, Germany, Catalogue No:0QIY), IL-6 (Human IL-6 ELISA BMS213/2CE Bender MedSystems GmbH, Vienna, Austria) and tumour necrosis factor alpha (TNF-α) (human TNF-immunoassay kit, BioSource International, Inc., Camarillo, California, USA) levels were also measured as markers of inflammation. Kt/V was used to estimate dialysis dose. The serum level of prohepcidin (Hepcidin Prohormone ELISA kit, DRG Instruments GmbH, Marburg, Germany) was determined by using a validated enzyme-linked immunosorbent assay (ELISA) kit. All blood samples were collected pre-dialysis with the exception of the post-dialysis serum urea nitrogen to calculate urea kinetics. The HCV antibody status was examined using the third generation of HCV enzyme immunoassay (EIA version 2.0; Abbott Laboratories, Abbott Park, IL).

Total iron received for each patient was defined as total iron treatment dose administered over the 12-months time period before the collection of serum samples for laboratory tests. The mean rhuEPO per week and the total rhuEPO dose during 12-month were also calculated.

### Statistical analysis

The statistical analysis was carried out by Statistical Package for Social Sciences for Windows ver. 15.0 (SPSS Inc., Chicago, IL, USA). Data are expressed as mean ± SD, with significance level p < 0.05. For dichotomous variables the frequency of positive occurrences were given along with their corresponding percentages. Median values and percentile 25 and percentile 75 were used to present the results of variables with a non-normal distribution. Parametric and nonparametric tests were used according to the distribution pattern of the data of each variable. Statistical comparisons of individual groups were based on Student’s *t* test for continuous variables and on Fisher’s exact test for discrete variables. All statistical tests performed were two sided and the level of significance was 0.05. In correlation analysis between numerical parameters for non-normal distributed variables, Pearson’s correlation test was used with transformed data. Multiple linear regression model was used to identify the independent determinants of outcome variable after adjustment for potential confounding factors for whole HD, HCV positive and negative groups.

## Results

### Patient baseline characteristics

The baseline characteristics of the 60 patients (30 patients in HCV positive group and 30 patients in HCV negative group) and 20 healthy volunteers were given in Table 1. The HD patients were older than the healthy volunteers. There were no differences in gender, body mass index (BMI), pre-dialysis systolic and diastolic blood pressure levels between HD patients and healthy controls. Among laboratory parameters, the serum concentrations of glucose, creatinine, triglycerides and phosphorus were significantly higher in the HD group than the healthy controls. Serum albumin, calcium and LDL-cholesterol levels were also significantly lower in the HD group (Table 1).

**Table 1:** Clinical and laboratory characteristics of the study population including healthy volunteers and maintenance hemodialysis patients

	**Healthy volunteers (n = 20)**	**HD patients (n = 60)**	***P value***	**HCV (+) patients (n = 30)**	**HCV (−) patients (n = 30)**	***P value***
***Clinical Characteristics***						
Gender (M/F)	13/7	27/33	*0.121*	14/16	13/17	*0.795*
Age (range), years	38 ± 8 (27–57)	50 ± 15 (18–82)	*<0.001*	47 ± 15 (25–82)	52 ± 15 (18–76)	*0.226*
BMI (kg/m^2^)	24.4 ± 2.7	24.1 ± 4.3	*0.766*	23.3 ± 3.3	24.9 ± 5.0	*0.145*
Systolic BP (mm Hg)	113 ± 10	119 ± 17	*0.231*	118 ± 18	121 ±20	*0.562*
Diastolic BP (mm Hg)	71 ± 8	74 ± 16	*0.378*	73 ± 19	76 ± 14	*0.441*
Time on HD (months)	-	80 ± 51	*-*	92 ± 58	68 ± 42	*0.072*
Diabetes, n (%)	-	2 (3.3%)	*-*	1 (3.3%)	1 (3.3%)	*-*
***Laboratory data***						
WBC count, 10^9^ cells/L	6.8 ± 1.4	6.6 ± 2.1	*0.766*	6.27 ± 2.18	6.91 ± 2.09	*0.293*
Haemoglobin (g/dL)	14.0 ± 1.4	11.7 ± 1.4	*<0.001*	11.6 ± 1.5	11.7 ± 1.5	*0.786*
Haematocrit (%)	40.9 ± 3.9	34.6 ± 4.5	*<0.001*	34.1 ± 5.1	34.7 ± 4.6	*0.855*
Fasting glucose (mg/dL)	80.7 ± 6.7	88.6 ± 16.0	*0.004*	89.2 ± 18.3	88.2 ± 14.6	*0.830*
Serum creatinine (mg/dL)	0.87 ± 0.19	8.81 ± 2.08	*<0.001*	8.50 ± 2.26	9.06 ± 1.87	*0.317*
Total cholesterol (mg/dL)	179 ± 44	165 ± 41	*0.170*	157 ± 38	170 ± 42	*0.235*
HDL-cholesterol (mg/dL)	36 ± 12	36 ± 12	*0.874*	38 ± 13	35 ± 11	*0.298*
LDL-cholesterol (mg/dL)	110 ± 33	91 ± 33	*0.039*	86 ± 28	94 ± 36	*0.374*
Triglycerides (mg/dL)	134 ± 61	185 ± 103	*0.040*	159 ± 65	205 ± 122	*0.080*
Albumin (g/dL)	4.51 ± 0.40	4.00 ± 0.41	*<0.001*	3.90 ± 0.46	4.10 ± 0.35	*0.078*
ALT (U/L)	28.9 ± 18.5	18.2 ± 14.5	*0.021*	24.8 ± 17.0	13.5 ± 10.3	*0.011*
Calcium (mg/dL)	9.2 ± 0.4	8.8 ± 0.8	*0.027*	9.0 ± 0.9	8.7 ± 0.8	*0.226*
Phosphorus (mg/dL)	3.3 ± 0.4	5.3 ± 1.3	*<0.001*	5.5 ± 1.4	5.3 ± 1.1	*0.577*

Abbreviations: HCV: hepatitis C virus, M: male, F: female, BMI: body mass index, BP: blood pressure, HD: hemodialysis, WBC: white blood cell, ALT: Alanine aminotransferase.

On comparing HCV positive and negative HD patients, there were no differences in age, gender, BMI, time on dialysis, biochemical parameters (serum glucose, creatinine, total cholesterol, LDL-cholesterol, HDL-cholesterol, triglycerides, total protein, albumin, calcium and phosphorus levels) and complete blood cell counts (leukocytes, hemoglobin, hematocrit) between HCV positive and negative groups (Table 1). The neutrophil/lymphocyte ratio was similar between HCV positive (2.02±0.64) and negative (2.79±2.27) groups (p = 0.30). As expected, serum concentrations of ALT were significantly higher in the HCV positive patients than HCV negative patients (p = 0.011).

### Inflammatory and iron parameters

The serum inflammatory and iron parameters of HD and control groups are shown in Table 2. Serum hs-CRP, IL-6, TNF-α and ferritin levels were significantly higher in the HD group than the controls (Table 2). Serum prohepcidin levels were similar between HD and control groups.

**Table 2:** Inflammatory and iron parameters in healthy volunteers and maintenance hemodialysis patients

	**Healthy volunteers (n = 20)**	**HD patients (n = 60)**	***P value***	**HCV (+) patients (n = 30)**	**HCV (−) patients (n = 30)**	***P value***
***Inflammatory parameters***						
hs-CRP (mg/L) median (25-75%)	1.83 (0.68-2.79)	8.35 (3.52-16.22)	***0.005***	**12.20 (5.07-21.42)**	**5.45 (3.25-1.57)**	*0.181*
TNF-alpha (pg/mL)	6.4 ± 4.6	68.0 ± 104.1	*0.026*	76.1 ± 61.0	61.0 ± 29.6	*0.593*
IL-6 (pg/mL)	3.5 ± 4.2	12.8 ± 10.1	*0.021*	9.1 ± 7.7	15.8 ± 10.9	*0.016*
***Iron parameters***						
Iron (μg/dL) median (25-75%)	78 (60–118)	71 (57–98)	*0.395*	88 (66–130)	64 (44–75)	*<0.001*
TIBC (μg/dL)	289 ± 93	247 ± 50	*0.156*	229 ± 54	263 ± 41	*0.012*
TSAT (%)	37.2 ± 24.2	35.1 ± 21.4	*0.771*	46.9 ± 24.1	24.7 ± 11.6	*<0.001*
Ferritin (ng/mL)	77 ± 76	705 ± 420	*<0.001*	673 ± 503	699 ± 270	*0.579*
Prohepcidin (ng/mL)	137 ± 20	142 ± 23	*0.417*	135 ± 25	148 ± 18	*0.025*
Total iron need (mg/year) median (25-75%)				0 (0–200)	150 (0–425)	*0.047*
**ESA resistance index**				0.03 (0.00-6.51)	**0.07 (0.00-6.58)**	*0.039*

Abbreviations: hs-CRP: high sensitive C-reactive protein, TNF-α: tumour necrosis factor alpha, IL-6: Interleukin-6, TIBC: total iron binding capacity, TSAT: Transferrin saturation, ESA: erythropoiesis-stimulating agent.

On comparing HCV positive and negative HD patients, prohepcidin levels of HCV positive patients (135 ± 25 ng/mL) were significantly lower than HCV negative patients [148 ± 18 ng/mL, (p = 0.025)], but similar to healthy controls [137 ± 20 ng/mL (p = 0.780)] (Table 2). Serum IL-6 levels of HCV positive patients were also significantly lower than HCV negative patients (p = 0.016) and higher than healthy controls (p = 0.023). Serum iron levels and TSAT were significantly higher in HCV positive patients (p < 0.001 and p < 0.001, respectively). Serum ferritin levels were similar between two groups. The total iron received over the 12 months period was significantly lower in the HCV positive patients [0 mg (0–200)] than the HCV negative patients [150 mg (0–425)] (p = 0.047). In HCV negative group the number of patients receiving rhuEPO (n = 17, 57%) was significantly higher than the HCV positive group (n = 7, 23%) (p = 0.008). The ESA resistance index values (median (25%-75%)) were also higher in the HCV negative patients [0.07 (0.00-6.58) vs 0.03(0.00-6.51) (p = 0.039)].

### Correlations

In the univariate correlation analysis, serum prohepcidin levels were positively correlated with ferritin (r = 0.405, p = 0.001) and IL-6 (r = 0.271, p = 0.050) levels in HD patients. The serum IL-6 levels were also correlated with ESA resistance index (r = 0.309, p = 0.027). Multiple linear regression analysis for predicting prohepcidin levels was performed including serum ferritin and IL-6 levels in model as possible confounding factors and revealed that serum ferritin levels were independently associated with prohepcidin levels (r = 0.45, standardized β=0.380, p = 0.007) in all HD patients.

In the HCV positive group, serum prohepcidin levels significantly correlated with time on dialysis (r = 0.428, p = 0.018) and ferritin levels (r = 0.514 p = 0.004). Serum levels of prohepcidin were not correlated with age, serum IL-6, TNF-α, hs-CRP, albumin, hemoglobin levels and ESA resistance index values. Serum IL-6 levels were only correlated with serum ferritin levels (r = 0.421, p = 0.046) and ESA resistance index values in the HCV positive group (r = 0.461, p = 0.27). Multiple linear regression analyses for predicting prohepcidin levels was performed including time on dialysis and serum ferritin levels in model as possible confounding factors and revealed that serum ferritin levels were independently associated with prohepcidin levels (r = 0.612, standardized β=0.628, p = 0.008).

**Figure 1:**
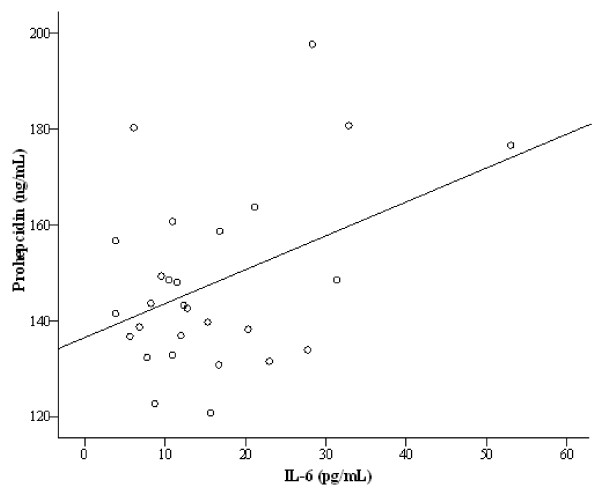
The correlation analysis between prohepcidin and IL-6 levels in HCV negative HD patients (r = 0.418, p = 0.027).

In the HCV negative group, serum prohepcidin levels significantly correlated with serum IL-6 levels (r = 0.418, p = 0.027), total iron dose (r = 0.373, p = 0.042) and ESA resistance index (r = 0.418, p = 0.021). Serum levels of prohepcidin were not correlated with age, serum TNF-α, hs-CRP, albumin, ferritin and hemoglobin levels in this group. When multiple regression analysis was performed including serum IL-6 levels, total iron dose and ESA resistance index in model as possible confounding factors, serum ferritin levels were found to be independently associated with prohepcidin levels (r = 0.680, standardized β=0.402, p = 0.032) (Figure 1).

## Discussion

In the present study, serum prohepcidin and IL-6 levels were found to be lower in HD patients with chronic HCV infection compared to HCV negative HD patients. To our knowledge, this is the first study to show that serum prohepcidin levels were significantly lower in HD patients with chronic HCV and that concentrations of prohepcidin were associated with serum ferritin levels. Growing evidence has suggested the potential occurrence of dysregulation of the hepcidin system in patients with chronic viral hepatitis. In a non uremic population, Nagashima and coworkers [14] have reported that serum prohepcidin levels were significantly lower in chronic HCV infection which may be associated with the down-regulation of hepcidin transcription by HCV induced reactive oxygen species [10]. There is convincing evidence that serum hepcidin levels were significantly lower in patients with hepatitis C [9,15]. However, Lee SH et al. showed increased serum IL-6 and prohepcidin levels in patients with hepatitis C [16]. Another study found that hepatic hepcidin expression levels in chronic liver diseases were strongly associated with the serum ferritin concentration [10]. These findings may explain the excess iron storage in patients with chronic HCV infection. However, the data in the literature on relation among hepcidin prohormone, iron parameters, inflammation and hepatitis C in HD patients are very limited. The relationships between iron stores and HCV infection, and low iron requirement in HD patients with HCV infection are reported previously [12]. In the present study, HCV positive patients also required lesser iron treatment and/or rhuEPO. Additionally, serum levels of iron and TSAT were found significantly higher in HCV positive HD patients compared to HCV negative patients. Similarly, lower serum prohepcidin levels were previously reported in patients with chronic HCV infection and concentrations of this molecule were negatively associated with total iron stores and contribute to the low rhuEPO resistance and the need for low doses of rhuEPO in these patients. [9-11]. Our results suggest that disordered hepcidin regulation could be another responsible factor for lower iron and rhuEPO requirements in HD patients with chronic hepatitis C.

In recent studies, serum levels of prohepcidin and hepcidin were found to be significantly higher in HD patients compared to healthy controls [7,8]. In the present study, we found no difference in serum prohepcidin levels between HD patients and controls. Due to the case control nature of the present study, half of the patients were HCV positive who had lower serum prohepcidin levels than the HCV negative patients. For that reason, the mean serum prohepcidin levels of the HD patients were similar to healthy controls. However, serum prohepcidin levels of HCV negative HD patients were significantly higher than the healthy controls, which is consistent with previous reports [7,8]. There may be several possible mechanisms of lower prohepcidin levels in dialysis patients with hepatitis C; 1) the down-regulation of hepcidin transcription by HCV induced reactive oxygen species, 2) impaired induction of hepcidin by IL-6 in the setting of chronic hepatitis C and 3) the down-regulation of hepcidin expression by erythropoietin via inhibiting hepcidin transcription in liver cells [7,8,17].

Inflammation is an important inducer of hepcidin synthesis [18,19]. In HD patients, inflammation is also a well known feature and actually in our study all the inflammation markers including hs-CRP, IL-6 and TNF-α were found higher compared to healthy controls. The causes of highly prevalent state of inflammation in HD patients are multiple, including decreased renal function, volume overload, comorbidity and intercurrent clinical events [20]. Additionally, in HD group, serum IL-6 levels were correlated with prohepcidin levels similar to observational data derived from the nonuremic population [18,19]. In the present study, although the correlation between serum IL-6 and prohepcidin levels were significant in HCV negative HD patients, no significant correlation was found between serum IL-6 and prohepcidin levels in HCV positive HD patients. These findings may indicate that the hepcidin response to inflammatory cytokines such as IL-6 may be inappropriate in HCV positive HD patients which may be one of the mechanisms responsible for dysregulation of hepcidin in HD patients with chronic HCV infection.

Hepatitis C virus infection may result in lower production of type 1cytokines including IL-6 [21,22]. The suggested possible mechanism for this finding is the impairment of monocyte function in patients with chronic hepatitis C [21]. In the present study, HCV positive HD patients had also lower serum IL-6 levels as compared to HCV negative patients. These lower serum IL-6 levels in HCV positive HD patients may be responsible for the decreased prohepcidin levels in HCV as compared to HCV negative HD patients.

In the present study, we measured serum prohepcidin levels, which is one of the limitations. The detection and quantification of hepcidin in serum have been hampered by several technical difficulties. However, *Costa et al.* reported significant positive correlation between prohepcidin and hepcidin serum levels [7]. There is also evidence that prohepcidin levels are reliable indicators of hepcidin levels and activity [23]. Because of the case control design of the study, the results can not infer a causal relationship. This is the second limitation of the study.

## Conclusions

HCV positive HD patients have low levels of serum prohepcidin and IL-6 which might account for iron accumulation together with lower iron and rhuEPO requirements in these patients.

## Competing interests

The authors declare that they have no competing interests.

## Authors’ contributions

YC have made substantial contributions to conception and design, acquisition of data, analysis and interpretation of data. BY participated in the design of the study and helped the collection of data. AO participated in the design of the study and performed the statistical analysis. NG participated in design and coordination of the study and helped the collection of data. HY participated in design and coordination of the study and helped the collection of data to draft the manuscript. AT carried out the biochemical studies. AY conceived of the study, and participated in its design and coordination and helped to draft the manuscript. All authors read and approved the final manuscript.

## Pre-publication history

The pre-publication history for this paper can be accessed here:

http://www.biomedcentral.com/1471-2369/13/56/prepub
